# Mesoporous Bioactive Nanoparticles for Bone Tissue Applications

**DOI:** 10.3390/ijms24043249

**Published:** 2023-02-07

**Authors:** Daniel Arcos, María Teresa Portolés

**Affiliations:** 1 Departamento de Química en Ciencias Farmacéuticas, Facultad de Farmacia, Universidad Complutense de Madrid, Instituto de Investigación Sanitaria Hospital 12 de Octubre i+12, Plaza Ramón y Cajal s/n, 28040 Madrid, Spain; 2 CIBER de Bioingeniería, Biomateriales y Nanomedicina, CIBER-BBN, ISCIII, 28040 Madrid, Spain; 3 Departamento de Bioquímica y Biología Molecular, Facultad de Ciencias Químicas, Universidad Complutense de Madrid, Instituto de Investigación Sanitaria del Hospital Clínico San Carlos (IdISSC), 28040 Madrid, Spain

**Keywords:** mesoporous nanoparticles, bioactivity, bone regeneration, drug delivery, therapeutic ions, cellular response, in vivo studies

## Abstract

Research in nanomaterials with applications in bone regeneration therapies has experienced a very significant advance with the development of bioactive mesoporous nanoparticles (MBNPs). These nanomaterials consist of small spherical particles that exhibit chemical properties and porous structures that stimulate bone tissue regeneration, since they have a composition similar to that of conventional sol–gel bioactive glasses and high specific surface area and porosity values. The rational design of mesoporosity and their ability to incorporate drugs make MBNPs an excellent tool for the treatment of bone defects, as well as the pathologies that cause them, such as osteoporosis, bone cancer, and infection, among others. Moreover, the small size of MBNPs allows them to penetrate inside the cells, provoking specific cellular responses that conventional bone grafts cannot perform. In this review, different aspects of MBNPs are comprehensively collected and discussed, including synthesis strategies, behavior as drug delivery systems, incorporation of therapeutic ions, formation of composites, specific cellular response and, finally, in vivo studies that have been performed to date.

## 1. Introduction

In recent decades, the development of new nanomaterials for the treatment of bone defects and bone-related pathologies has experienced significant advances. Particularly, inorganic nanoparticles have shown great potential as nanosystems for diagnosis, drug delivery, magnetic hyperthermia, photothermal therapy, and gene transfection, and have provided new research expectations as well as new strategies for clinical practice [1,2,3,4]. In the field of bone defects and pathologies, bioactive mesoporous nanoparticles (MBNPs) have been an important advance in the last 15 years, contributing significantly to the improvement of implantable devices, such as coatings for endosseous prostheses, macroporous scaffolds, bone cements, etc. [5].

Mesoporous bioactive glasses (MBG) were first proposed by Yan et al. in 2004 [6] and can be considered the immediate precedent of MBNPs. MBGs are bioceramics, generally prepared in the SiO_2_-CaO-P_2_O_5_ system, which exhibit high porosity and specific surface area values. Because of these textural properties, MBGs exhibit superior bioactive behavior to bioactive glasses prepared by more conventional methods such as melting or sol–gel methods. MBGs are commonly prepared by hydrolysis and condensation of SiO_2_, P_2_O_5_, and CaO precursors in the presence of amphiphilic molecules, which act as structure-directing agents (SDA). This gives rise to a system of highly organized micelles, on which the inorganic precursors form the glassy component using the SDA micelles as a template. By removing the SDA by calcination or extraction, bioactive glasses with an ordered porous system in the mesopore range (2–50 nm) are obtained [7].

Shortly after the discovery of MBGs, interest arose in preparing these materials in the form of small particles, now known as bioactive mesoporous nanoparticles (MBNPs). The different low-temperature synthesis methods that had been developed, together with the versatility to incorporate new chemical elements [8,9], offered excellent possibilities to develop nanosystems for delivery of therapeutic agents capable of stimulating bone regeneration and treating pathologies associated with bone defects, such as osteoporotic fractures, bone tumors, and osteomyelitis.

**Figure 1:**
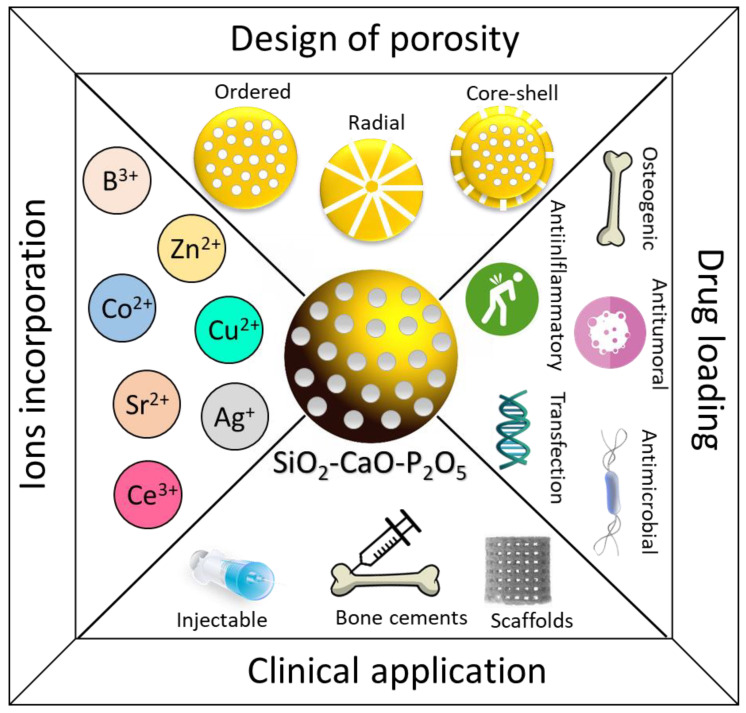
Schematic representation of the different strategies used to optimize the biological behavior of mesoporous bioactive nanoparticles.

In the present work, a comprehensive review of the most relevant literature published in the last 15 years has been carried out, in which the different advances in the topic of MBNPs for bone tissue applications are critically analyzed and discussed. These topics include the most significant synthesis routes, the ways of integrating MBNPs into different biomedical devices, the use of MBNPS as drug and therapeutic ion delivery systems, and, finally, studies of specific cellular responses to MBNPs and the in vivo studies performed so far (Figure 1).

## 2. Synthesis Strategies for the Preparation of Mesoporous Bioactive Nanoparticles

Interest in obtaining bioactive glasses in the form of nanoparticles has been increasing in the recent decades due to their potential applications in bone tissue regeneration, soft tissue applications, and wound healing. Moreover, the development of sol–gel chemistry has provided new possibilities for the preparation of bioactive glass nanoparticles, which have been added to the conventional melt–quench approach. For more information on these techniques, we recommend the excellent reviews by Zheng et al. [8] and Vichery et al. [9]. Prof. Stucky’s group first carried out the synthesis of mesoporous bioactive spherical particles with nano and/or micrometer size in 2006 [10]. These authors proposed the preparation of mesoporous spheres in the SiO_2_-CaO-P_2_O_5_ system by aerosol-assisted methods using the triblock copolymer P123, which had been previously used as a structure-directing agent in the synthesis of mesoporous bioactive glasses. Prof. Stucky’s group proposed that these spheres could have two applications. The first was as a bone substitute, by including them in injectable cements, due to their enhanced rates of hydroxyapatite deposition compared to irregular particles. The second application was as a fast-acting hemostatic agent, i.e., as a compound to be poured into a wound to stabilize the victim before bleeding to death. Both properties are based on the high specific surface area and porosity of the mesoporous spheres, as well as the role of Ca^2+^ ions, through the cascade of reactions leading to polymerization and stabilization of the blood clot.

**Figure 2:**
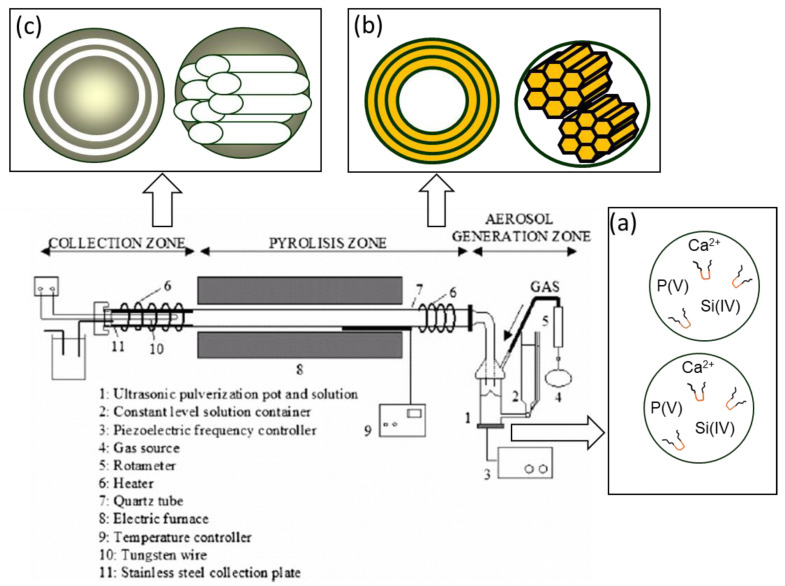
Scheme of an aerosol-assisted method device for the synthesis of mesoporous bioactive nanoparticles: (**a**) aerosol droplets containing surfactant molecules and inorganic precursors in the aerosol generation zone; (**b**) aerosol droplets containing hexagonal and/or lamellar self-assembled micelles at the heating zone; (**c**) mesoporous bioactive nanoparticles collected after the pyrolysis process. Additional heat treatment is required to completely remove the organic component.

**Figure 3:**
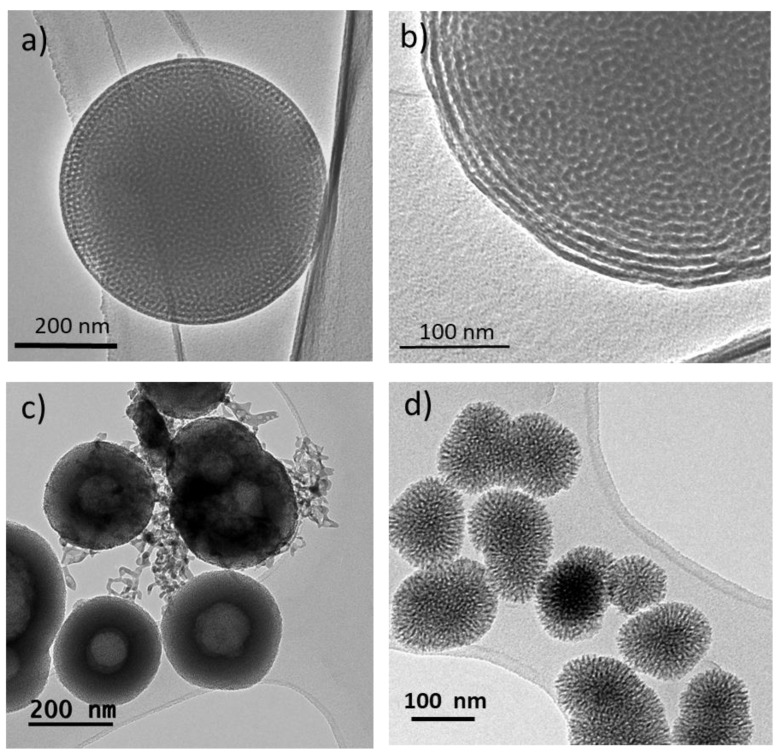
Different examples of MBNPs prepared by different methods. Hexagonal ordered (**a**) and lamellar (**b**) mesoporous nanoparticles prepared by aerosol-assisted pyrolysis; (**c**) core–shell MBNPs prepared by the dual-templated method; and (**d**) radial mesoporous MBNPs prepared by biphasic stratification.

Aerosol-assisted methods have the advantage of being able to control the properties of the spheres by means of the flow of the carrier gas, concentration of the precursor solution, frequency of the ultrasound used to generate the aerosol, etc. (Figure 2). Despite this, it is difficult to achieve homogeneous particle sizes, and, usually, polydisperse particle sizes with diameters between 0.1 and 3 μm are obtained. In this context, it should be noted that, as the particle size increases, it is possible to obtain highly ordered mesoporous phases, especially when surfactants with longer hydrophilic components are used (Figure 3). Thus, Arcos et al. [11] were able to obtain bioactive mesoporous microspheres with a hexagonal mesoporous structure using the aerosol-assisted method and Pluronic F127 as a template. These microspheres were shown to have the ability to load and release triclosan, exhibiting a potential application to be used in periodontal regenerative surgery and infection prophylaxis.

The synthesis of bioactive mesoporous nanospheres with sizes between 100 to 200 nm and a narrow particle size distribution can be obtained by the sol–gel method under alkaline conditions. Similar to the preparation of mesoporous SiO_2_ nanoparticles by the popular modified Stöber method [12], MBNPs in the SiO_2_-CaO-P_2_O_5_ system can be obtained from the corresponding silicon and phosphorus alkoxides and inorganic calcium salts, which hydrolyze and condense in the presence of a catalyst of alkaline nature. In this context, several authors have proposed the preparation of nanoparticles in the SiO_2_-CaO-P_2_O_5_ system with different structure agents, giving rise to mesoporous nanoparticles with different mesoporous structures (Figure 3) such as disordered [13], dual hollow core–shell structures [14], etc. (Table 1). Several authors highlight the ease of this method, which is based on the formation of micellar structures using a SDA in an aqueous alkaline medium, to later add the different precursors in a controlled manner. The polycondensation of the hydrolyzed precursors on the hydrophilic fraction of the micelles determines the mesoporous structure. On the other hand, the formation of spherical and monodisperse SiO_2_ nanoparticles is quantitatively understood in terms of the stabilization of the surface energy, i.e., the competition generated from the nucleation and growth processes. At high pH, the particles are negatively charged on their surface, and this creates stability against aggregation in the sol. A narrow particle size distribution is indicative that the nucleation process is limited to the early stages, where the monomer reacts preferentially with nucleated particles that serve as seeds [15]. However, although the properties of mesoporous SiO_2_ particles are easily controllable through the many strategies developed so far [16], the complexity of the SiO_2_-CaO-P_2_O_5_ ternary system makes it questionable whether the porous structure, dispersion, and even chemical composition can be easily controlled. For example, in the pioneering paper by Yun et al. [17], the hexagonal mesoporous order of mesoporous SiO_2_ nanoparticles is transformed into a worm-like structure as a function of CaO content. One of the most serious disadvantages of this method is the limitation in the CaO content that can be incorporated, since the bioactive behavior and, therefore, the osteogenic capacity of these compounds is highly dependent on the CaO content. In early attempts to synthesize MBNPs by this method [17,18] the nominal composition was 80% SiO_2_, 16% CaO, and 4% P_2_O_5_ (molar %), although the experimental composition was not reported, as the authors did not perform elemental chemical analysis on their MBNPs. Subsequently, Hu et al. [19] synthesized hollow MBNPs, demonstrating that by varying the amount of hexadecyltrimethyl ammonium (CTAB) they could control the shell thickness. According to these authors, this is because the kinetics of TEOS hydrolysis is dependent on the concentration of CTAB. However, the CaO content that could be determined by EDX spectroscopy was only 11% (molar %) without demonstrating in vitro bioactivity of these nanoparticles. More recently, Ding et al. [14] have also prepared hollow MBNPs by controlling their size, morphology, and structure by varying CTAB concentrations, obtaining MBNPs with loading capacity and vancomycin hydrochloride release. Similarly to the previous authors, the nominal composition had low CaO content (80SiO_2_-15CaO-5P_2_O_5_) without the authors studying the true chemical composition by elemental chemical analysis.

An interesting synthetic route is the one proposed by El-Fiqi [20] based on the ultrasound-assisted sol–gel method. In this way, the preparation of hollow particles with an inner cavity large enough to accommodate proteins is achieved. In this case, a high-volume SDA such as PEG is needed, which, after removal, leaves a large interior cavity and a mesoporous shell. However, the limitation on incorporating high-CaO contents persists. In this work, the theoretical composition of MBNPs was 85 SiO_2_-15 CaO (% mol), which is equivalent to a Ca/Si ratio of 0.176. Elemental chemical analysis showed that the Ca/Si ratio in the obtained MBNPS was 0.157 ± 0.013. A very interesting aspect of this work is the study that the author performed by XPS showing that the Ca/Si ratio on the surface of the MBNPs was 0.29 (considering a penetration of up to 10 nm). This implies a CaO concentration almost twice as high on the surface compared to the interior of the nanoparticles, which could justify the ability of these nanosystems to form an apatite-like phase similar to the mineral component of bone on their surface, when reacted with simulated body fluid (SBF).

One of the most interesting strategies for the preparation of MBNPs with high capacity for drug loading and release is the use of the dual-template system [21]. This method offers wide versatility to design different mesoporous structures by combining two SDAs of different sizes and/or chemical natures. In this way, MBNPs can be designed with different pore distributions in the interior and in the shell, providing higher control over the drug release, which is generally limited by the size and porous structure of the shell. Wu et al. [22] used this method to obtain spherical MBNPs with diameters between 50–100 nm using a hydrothermal method and CTAB and PVP as structure-directing agents. In this way, they obtained MBNPs that showed apatite mineralization in contact with SBF and the ability to release doxorubicin hydrochloride, an anti-cancer drug. These authors proposed the synthesis of these MBNPs in the SiO_2_-CaO-P_2_O_5_ system using the classical precursors tetraethylortosilane (TEOS), calcium nitrate, and triethylphosphate (TEP) with a theoretical Ca:P:Si molar ratio of 15:5:80. However, Wu et al. highlighted one of the limitations that, beyond the difficulty of incorporating CaO in amounts higher than 15%, has been observed in the preparation of MBNPs, i.e., the difficulty for phosphorus to be incorporated into the inorganic matrix of SiO_2_-CaO-P_2_O_5_ MBNPs. Indeed, these authors reported the absence of phosphorus in the final composition, despite incorporating it in the form of TEP, as was subsequently observed by other research groups [23,24].

Li et al. [24] developed an interesting synthetic strategy based on two structuring agents, i.e., diblock copolymer PS-b-PAA and the cationic surfactant CTAB. To carry out the synthesis of MBNPs in the SiO_2_-CaO-P_2_O_5_ system, PS-b-PAA is dissolved in tetrahydrofuran, while CTAB is dissolved in acidic medium under alkaline conditions. When both solutions are mixed and the inorganic phase precursors are added, a double template is formed, in which PS-b-PAA forms micelles that constitute the core of the particles, and CTAB interacts in such a way that it forms a shell with a radial mesoporous structure. This method resulted in MBNPs with a uniform diameter of about 250 nm, where the inner cavity was about 150 nm and was composed of pores of about 20 nm, because of PS-b-PAA decomposition. On the other hand, the radial mesoporous shell was 50 to 60 nm thick, with radial mesopores templated by CTAB of about 2.6 nm. These MBNPs, despite not incorporating the added phosphorus in the form of TEP, had an approximate composition of 79 SiO_2_-21 CaO (% mol) and exhibited apatite-forming capacity on their surface as well as doxorubicin loading and release. This CaO content is one of the highest that has been achieved in the preparation of MBNPS by precipitation methods. Subsequent work carried out by our research group showed that the incorporation of CaO is facilitated by the presence of the phosphorus precursor TEP in the reaction medium, although the presence of the latter element cannot be detected in the final composition [23].

The preparation of microemulsions using different SDAs allows control of the final particle size and even particle morphology. Liang et al. [25] were able to prepare spherical MBNPs with radial mesopores using CTAB and ethylacetate to form a microemulsion. As a function of temperature, they were able to prepare spherical MBNPS with radial porosity by precipitating the nanoparticles at room temperature, while they obtained pineal morphology MBNPs with lamellar mesoporous structure by treating the microemulsion by hydrothermal methods.

In order to increase the CaO and P_2_O_5_ content in MBNP_S_, some authors have proposed the prior preparation of mesoporous SiO_2_ or SiO_2_-P_2_O_5_ nanoparticles followed by impregnation in dissolved calcium nitrate and subsequent calcination at temperatures of 600 °C or higher. In this context, Han et al. [26] reported a method for the synthesis of SiO_2_-CaO MBNPs with a dendritic porous structure based on the use of CTAB and sodiumdodecylsulfate (SDS), presenting the particularity that this method is carried out without organic solvents. These authors initially precipitated mesoporous dendritic SiO_2_ nanoparticles and incorporated the CaO by subsequent impregnation in a calcium nitrate solution. Similarly, Xie et al. [27] fabricated radial mesoporous SiO_2_-P_2_O_5_ nanospheres using CTAB as a structure-directing agent and triethanolamine (TEA) as a hydrolysis catalyst. Solid reactions were then carried out to synthesize SiO_2_-CaO-P_2_O_5_ MBG using SiO_2_-CaO nanoparticles as silicon and phosphorus sources and a solution of Ca(NO_3_)_2_ as the calcium source, which was subsequently calcined to remove nitrates and incorporate Ca^2+^ to the glassy network. The prepared MBG not only displayed the radial structure and high specific surface area (~321 m^2^/g), but also had high phosphorus and calcium content.

Most of the synthetic strategies for the preparation of MBNPs are based on sol–gel chemistry in the presence of one or more soft structure agents. In addition to the described routes, other routes have been developed that incorporate variants such as two-step acid-catalyzed sol–gel synthesis, selective etching and impregnation, etc. (see Table 1). However, there are alternatives to the use of soft templates where hard inorganic materials such as calcium carbonate serve as molds for the preparation of hollow core–shell nanoparticles [28].

**Table 1:** Synthesis methods and characteristics of mesoporous bioactive particles.

Synthesis Method	Particle Size and Morphology	Structure Directing Agent ^1^	Mesoporous Structure	Pore Size (nm)	Ref.
Aerosol-assisted	Polydisperse 0.1–1 μm	P123	Worm-like	6.5–6.9	[10]
	Polydisperse 0.2–3 μm Spherical	F127 P123 CTAB	Hexagonal Worm-like N/A	3.5 3.9 3.7	[11]
Ultrasound-assisted sol–gel	117 nm Spherical	PEG	Hollow core + Porous shell	7 (shell)	[20]
Sol–gel in alkaline conditions	20–200 nm Spherical	CTAB	Worm-like	1.8–2.2	[17]
	560 nm	DDA	N/A	2.4	[18]
	187–294 nm Spherical	CTAB	Hollow core + porous shell	4.6–8.8	[19]
	24 nm Spherical	CTAT	Disordered	9.7	[13]
	180–280 nm Spherical	CTAB	Hollow core + porous shell	5–7	[14]
Dual soft template	250 nm Spherical	PS-b-PAA + CTAB	Hollow core + porous shell	Bimodal 20 (core) 2.6 (shell)	[24]
	133–254 nm (Spherical shape) 28–193 nm (Pineal shape)	EA + CTAB	Radial Lamellar	Bimodal 5.1–14.0	[25]
	100 nm Spherical	P123 + CTAB	Worm-like	Bimodal < 2–3.7	[21]
	<100 nm Spherical	CTAB + SDS	Dendritic	Bimodal 3.7–16.4	[26]
	50–100 nm spherical	CTAB + PVP	Disordered	2.9	[22]
Two step acid-catalyzed sol–gel	200–250 nm Spherical	P123	Chanel-like	2.96	[29]
Selective etching and impregnation	400 nm Spherical	CTAB	Hollow core + mesoporous shell	2.7	[30]
Biphasic stratification followed by Ca impregnation	50–143 nm Spherical	CTAB	Radial	6.5–9.5	[27]
Biphase delamination	150–190 nm Spherical	CTAB	Radial	Bimodal 5–18	[31]
Hard template bioinspired	30–60 nm Spherical	Calcium carbonate	Hollow core + mesoporous shell	28.8	[28]
Inverse opal template	200–400 nm Spherical	F127 OMP OMC	Ordered mesopores Core–shell Open surface pores Disordered	10	[32]

^1^ P123: [(ethylene oxide, EO)20 (propylene oxide, PO)70 (EO)20]; F127: [(EO)106(PO)70(EO)106]) CTAB: hexadecyltrimethyl-ammonium bromide); SDS: sodiumdodecylsulfate; PEG: polyethylene glicol; DDA: dodecylamine; CTAT: hexadecyltrimethylammonium p- toluene sulfonate; PS-b-PAA: poly(styrene-b-acrylic acid); PVP: poly(vinylpyrrolidone); EA: ethyl acetate OMP: ordered macroporous polystyrene, OMC: ordered macroporous carbon.

## 3. Composites Based on Mesoporous Bioactive Nanoparticles

The main purpose of a composite material is the combination of a minimum of two materials with different physical and chemical properties to create a new material with better characteristics than those of the individual components to perform a specific function [33]. Among the different composite materials for bone tissue applications, biopolymer-based hydrogels that incorporate MBNPs are of particular interest to stimulate bone regeneration as injectable biomaterials or scaffolds. On the one hand, hydrogels have been used in numerous biomedical applications, such as tissue engineering [34], as they can mimic the structure of the extracellular matrix (ECM) by providing the appropriate microenvironment for cell adhesion and proliferation, as well as promoting the transfer of nutrients and metabolites [35]. Gelatin and alginate are biocompatible, non-immunogenic, and biodegradable natural hydrogels with excellent characteristics for scaffold design to repair damaged organs and tissues [36]. Besides, MBNPs have physical–chemical properties that make them ideal multifunctional nanosystems, due to their high biocompatibility, bioactivity, easy functionalization, and well-ordered internal mesopores with great loading capacity for many different types of therapeutic agents. For all these reasons, numerous recent studies have focused on the design of new composite materials based on the incorporation of MBNPs in hydrogels to promote mineralization [37] and to induce early osteogenic differentiation [38].

Currently, smart bio-inks are being developed for the preparation of scaffolds for bone repair with improved mechanical properties [39] and with the ability to guide cell growth, mimicking the excellent qualities and dynamics of natural ECM, to reproduce the microenvironment of bone tissue [40]. In this context, the incorporation of MBNPs in hybrid gelatin/oxidized chondroitin sulfate hydrogels has been demonstrated to enhance crosslinking and to accelerate the gelation, optimizing the mechanical features without affecting the injectability of the hydrogels and promoting the osteogenic differentiation of rat bone marrow mesenchymal stem cells in vitro and rat cranial defect restoration in vivo [41].

Particularly, the use of MBNPs doped with different ions as biochemical signals has produced great interest in specific bone regeneration. These nanoparticles can deliver biologically active ions such as calcium and silicon to create a microenvironment that stimulates osteogenic differentiation [42]. It is important to highlight the necessary coupling between the processes of osteogenesis and angiogenesis during bone repair. In this context, a very recent study demonstrated that osteogenic differentiation of primary mouse bone marrow stromal stem cells and angiogenesis were promoted by bioprinted alginate dialdehyde–gelatin/amine-functionalized copper-doped MBNP scaffolds without additional growth factors in vitro. The observed osteogenic and angiogenic effects were probably due to ionic stimulation by the incorporated copper-doped nanoparticles in this nanocomposite and to the increased cellular mechanosensitivity in the dynamic matrix [43]. Thus, these advanced bio-inks based on the incorporation of mesoporous bioactive glass nanoparticles in hydrogels have been proposed as a versatile strategy to generate cell-laden bioprinted scaffolds with enhanced bioactivity and biocompatibility as well as with osteogenic/angiogenic effects and with important bio-functional effects due to Ca^2+^, and Cu^2+^ ions as well as Si(IV) species.

Many studies on the design of composite biomaterials for osteogenic implants have employed various biologically active factors as osteogenic inducers, although these agents may present various side effects. In a recent study, silk fibroin was used as a scaffold biomaterial in combination with MBNPs as sustained release carriers for the Chinese drug icariin (ICA) in order to promote bone formation [44]. ICA is an active natural flavone glycoside extracted from Epimedium that promotes bone formation by osteoblasts and inhibits bone resorption by osteoclasts [45]. The obtained results evidenced that ICA and ions released from these scaffolds synergistically promote cell proliferation and differentiation of bone-marrow-derived mesenchymal stem cells.

More recently, osteogenic scaffolds were made by 3D printing employing nanocomposites of alginate dialdehyde–gelatin hydrogel reinforced with MBGNs and two types of ICA loading either into the nanoparticles or freely distributed within the hydrogel [46]. The nanoparticles improved the mechanical properties of the constructs and induced an apatite layer formation on the scaffolds, promoting cell adhesion and proliferation. The nanocomposite constructs delivered ICA efficiently, enhancing osteoblast proliferation, adhesion, and differentiation. The ICA release profile and kinetics in these composites could be modulated by tailoring drug loading into MBGNs in order to the avoid adverse effects of a high concentration of ICA.

Innovative studies on dental materials are also focused on composites based on mesoporous bioactive nanoparticles, MBNPs. MBNPs have the capability to remineralize enamel and dentin [47] with high bioactivity, lower cytotoxicity to dental pulp stem cells, and show antibacterial activity against intraoral bacteria [48]. In addition, resin and adhesives containing these nanoparticles, which release calcium and phosphate, improve the mechanical properties of demineralized hard tissues and reduce the presence of dental caries within areas where hygiene is difficult to maintain.

In this context, mesoporous bioactive glass nanoparticle/graphene oxide composites have recently been developed to study their effects on the mineralization ability and differentiation potential of human dental pulp stem cells (hDPSCs). These new composites promoted the differentiation of hDPSCs into odontoblast-like cells through the Wnt/β-catenin signaling pathway and are potential inducers of dentin formation [49].

## 4. Mesoporous Bioactive Nanoparticles as Drug Delivery Systems

The mesoporous structure of MBNPs makes these nanomaterials excellent candidates for use as drug carrier matrices. Thus, the bioactive behavior of MBNPs is reinforced by an additional functionality provided by the incorporated drug. Generally, drugs incorporated into MBNPs are used for the treatment of bone pathologies, which are often the cause of the bone defect they are intended to treat. On the other hand, MBNPs can be incorporated into 3D scaffolds, functionalized, or simply used as injectable material in the bone region to be treated.

### 4.1. Delivery of Osteogenic Agents

Enhancing the intrinsic osteogenic activity of MBNPs with osteoinductive drugs has been one of the most successful lines of research. El-Fiqi et al. [50] have studied the effects of dexamethasone incorporation into MBNPs, which were part of electrospun fibrous scaffolds, under in vitro and in vivo conditions. Dexamethasone (DEX) is a long-acting fluorinated corticosteroid useful in processes requiring anti-inflammatory and immunosuppressive treatment, but which has also demonstrated osteogenic properties [51]. These authors observed that the incorporation of MBNPs into electrospun polycaprolactone–gelatin fibers increased the tensile strength, elasticity, and hydrophilicity of the fibers. Moreover, the prepared MBNPs were capable of loading up to 63 wt% of DEX. After incorporation into the polymeric fibers, a sustained and almost linear release was achieved for 28 days, stimulating the proliferation and differentiation of stem cells derived from periodontal ligament. In vivo studies were first performed in a subcutaneous rat model, demonstrating the biocompatibility of the fibrous scaffolds. Finally, the scaffolds were implanted in a rat calvarial defect model. The DEX-loaded nanocomposite group showed significantly higher levels of bone volume and bone surface density compared to the scaffolds without DEX. DEX release from the fibrous nanocomposite scaffolds was found to stimulate bone formation processes in terms of the quantity and quality of the neo-bone structure.

Tissue regeneration processes require the action of multiple molecules in a time-dependent manner. Therefore, there is great interest in the development of sequential release systems of two or more drugs to facilitate these processes. Kang et al. [52] designed electrospun scaffolds of polyethylene oxide-polycaprolactone core–shell fibers containing SiO_2_-CaO MBNPs. The scaffold acted as a delivery system for two growth factors, FGF2 and FGF18. The former was incorporated into the core–shell fiber and was the first to be released, whereas FGF18 was incorporated into the MBNPs and released in a more sustained manner, thus achieving a sequential release of both growth factors. In vitro studies with rat mesenchymal stem cells showed that the system stimulated cell proliferation, alkaline phosphatase induction, and mineralization. Finally, in vivo studies in a calvaria model demonstrated that scaffolds loaded with both factors exhibited significantly higher bone-forming capacity.

Co-release of therapeutic ions with drugs from MBNPs has been proposed by Lee et al. [53] for the Sr^2+^ ion-Phenamil system, aiming to achieve a synergistic effect favoring bone tissue mineralization. Sr^2+^ has been shown to have important effects on hard tissue repair processes, while Phenamil has demonstrated activity as a potent BMP signaling activator molecule. Sr^2+^ ions were structurally incorporated into the chemical composition of MBNPs, obtaining in this case a composition 85Si:10Ca:5Sr (molar ratio), while phenamyl was incorporated externally into the porosity of MBNPs. These MBNPs were internalized into mesenchymal stem cells, and co-release of Sr^2+^ and phenamyl evidenced very significant synergistic effects on osteo/odontogenic gene expression, alkaline phosphatase activity, and cell mineralization in cell cultures, as well as osseous/dentinal regeneration in vivo. The authors concluded that the synergistic effect between Sr^2+^ and phenamyl occurs through the Trb3-dependent BMP signaling pathway.

### 4.2. Delivery of Antitumoral Drugs

Malignant bone tumors are a major factor in the loss of bone mass and in the formation of bone defects unrelated to trauma. Treatment of these diseases includes removal of tumor cells and restoration of lost bone mass. For these reasons, bioactive materials with inherent ability to stimulate bone healing and simultaneously kill cancer cells have been considered for different anticancer treatments, such as stimuli-responsive drug release [54] or magnetic hyperthermia treatments [55,56,57]. In this regard, MBNPs have demonstrated an inherent osteogenic capacity due to their chemical composition and textural properties as well as their ability to load and release drugs. In addition, MBNPs are easily injectable at the tumor site and can be phagocytosed by cancer cells provided they are small enough.

Doxorubicin is one of the most studied antitumor drugs to be loaded into MBNPs, demonstrating that it can be incorporated in very high amounts by controlling the pH of the loading medium. Wu et al. [22] managed to load up to 90% by weight of DOX into MBNPs prepared by the hydrothermal method, being able to control the amount of DOX incorporated by adjusting the initial drug concentration, while the release kinetics could be controlled by the pH of the surrounding medium. Similarly, Li et al. [24] were able to demonstrate that DOX could be incorporated in amounts greater than 80% by mass in hollow mesoporous bioactive glass spheres. Wang et al. [58] were able to load up to 63% and Firuzed [13] 35% into the MBNPs that these authors prepared using CTAT as a structure-directing agent.

Sui et al. [59] evaluated the ability of dendritic mesoporous bioactive nanospheres loaded with DOX to suppress tumors under in vivo conditions. Although this study does not contemplate the use of MBNPs for the treatment of bone tissue regeneration, the activity that these authors attribute to Ca^2+^ ions opens new possibilities for the use of MBNPs beyond the treatment of hard tissues. These nanoparticles act through a dual mechanism for the suppression of xenograft tumors induced in mice by injecting S180 tumor cells. On the one hand, calcium release in the intratumoral acidic environment activates transient receptor potential channels and calcium-sensing receptors in tumor cells, mediates calcium influx, and directly regulates the calpain-1-Bcl-2-caspase-3 signaling pathway to specifically suppress tumor growth without affecting normal cells. Moreover, the controlled release of DOX, which could be incorporated in amounts up to 43 wt%, improved antitumor efficacy, and its synergistic effect with Ca^2+^ ions reduced systemic toxicity.

Nawaz et al. [60] have also proposed the potential use of MBNPs for treatment of tumors other than bone tumors. In a recent work, these authors loaded silibinin (a natural flavonolignan used as an anticancer and tumor suppressor) onto MBNPs. Silibinin loading reached up to 63% by mass, and the system showed chemotherapeutic potential against metastatic MDA-MB-231 breast cells. The authors suggest that silbinin-loaded MBNPs can be applied as an effective delivery system to provide a chemopreventive response with potential application for breast cancer treatment.

The change in pH is one of the most important factors regulating the release of some antitumor drugs [61]. This fact is of particular importance since the environment of many tumors is significantly more acidic than that of healthy tissues. Shoaib et al. [62] published a preparation of MBNPs loaded with imatinib. This system achieved drug loading of 77.59%, demonstrating that the release kinetics of imatinib could be controlled by modifying the pH range between 4.4 and 10. These MBNPs demonstrated a significant inhibitory effect on the viability of MG-63 osteosarcoma cells, while supporting the formation of hydroxycarbonate apatite on their surface. This dual behavior suggests that these systems could be potentially used for the treatment of bone tumors and the subsequent regeneration of lost bone mass.

### 4.3. Delivery of Antimicrobial Drugs

One of the most serious complications of orthopedic prostheses in terms of morbidity, mortality, and medical costs is peri-implant infection. Although its incidence has decreased significantly due to antibiotic prophylaxis protocols, there is still a marginal risk of 0.5–5%, which implies that thousands of prostheses and other orthopedic devices become infected each year [63,64]. This is an increasingly important issue due to the larger number of patients undergoing prosthetic replacement surgery. Once bacteria adhere to the surface of the implant surface, they form a biofilm that provides resistance to antibiotics, becoming the main pathogenic factor of chronic infections. In fact, one of the milestones in this field of research is the development of nanostructured implant surfaces that decrease bacterial adherence and biofilm formation [65]. For this reason, one of the priority lines of research in the field of MBNPs is the development of dual systems that prevent bone infection while stimulating tissue regeneration, either to restore a bone defect or to stimulate peri-implant osteogenesis. To this end, antimicrobial drug loading against Gram+ and Gram- bacteria has been of interest to different research groups.

In 2009, Arcos et al. prepared triclosan-ordered bioactive microspheres using an aerosol-assisted method [11]. Triclosan diffuses through the bacterial cytoplasmic membrane and interferes with its lipid metabolism. At normal doses, it acts as a biocide, and at lower doses it has a bacteriostatic effect. Three series of microparticles differing in mesoporous structure were synthesized using different structure-directing agents (CTAB, P123, and F127), showing that the release kinetics of triclosan depended on the mesoporous structure formed by the different surfactants. These mesoporous particles showed a great capacity to form an apatite-like layer in contact with SBF, showing interesting characteristics to be used for bone regeneration and infection prophylaxis, although no antibacterial activity test was carried out by these authors. Moreover, the bioactive mesoporous particles prepared showed an excessively broad size distribution with too many large diameters (0.2–2 μm) to have the advantages of nanoparticles in terms of injectivity and cellular internalization. El-Fiqi et al. prepared MBNPs with a particle size ranging from 80–90 nm, which were able to load and release ampicillin sodium and siRNA, but, despite the more appropriate particle size, no antibacterial evidence was shown in this work either [66].

Fan et al. [67] carried out a very interesting work in which they demonstrated the potential of ampicillin-loaded MBNPs for dental applications, more specifically as a potential intra-channel disinfectant carrier. These authors showed that these nanosystems had the capacity to penetrate the dentin tubules, release the antibiotic, and present antibacterial and mineralization capacity under in vitro conditions. The MBNPs penetrated into the interior of dentin tubules obtained from sheep molars by ultrasound activation, where they were able to form apatite and reduce the proliferation of *Escherichia coli*.

More recently, important advances have been made in the design of antibiotic-loaded MBNPs for the treatment of infected bone defects, so that they stimulate bone regeneration while fighting infection. In this regard, MBNPs loaded with cinnamaldehyde have been proposed that have shown antibacterial activity against *Staphylococcus aureus* and *Escherichia coli* [68] as well as MBNPS loaded with Boswellia sacra extract [69] and vancomycin [14] that have exhibited in vitro antibacterial activity against *Staphylococcus aureus*.

### 4.4. Delivery of Anti-Inflammatory Drugs

In the field of bone implants, an added problem to pathogen action is osteolysis mediated by the inflammatory response associated with infection. There are numerous cellular secretion products that can adversely affect bone turnover. These include inflammatory cytokines IL-1, IL-6, PGE2, and TNF-α, among others, which are secreted into the environment by the action of macrophages as they phagocytize degradation products. These cytokines act as biochemical mediators by activating the receptor activator of nuclear factor kappa-B ligand (RANKL) and decreasing osteoprotegerin, resulting in an increase in osteoclastic activity and a decrease in osteoblastic activity with consequent loss of bone tissue [70]. Certainly, inflammatory cytokines secreted by immune cells contribute to restore tissue homeostasis and initiate the healing process. However, when the production of inflammatory cytokines becomes excessive, the inflammation remains unresolved and inflammatory disorders arise. In this context, the local release of anti-inflammatory agents from MBNPs has aroused the interest of different research groups to stimulate bone regeneration while avoiding situations of chronic inflammation.

Frequently, anti-inflammatory drugs have been used as model drugs to evaluate the capacity of MBNPs as drug carrier matrices, as well as to study the release kinetics as a function of the properties of the matrix [71,72,73]. In this context, MBNPs loaded with ibuprofen have been prepared, where it has been demonstrated that the drug release kinetics are closely related to the CaO content of the nanoparticles based on the binary composition SiO_2_-CaO [72].

Wang et al. performed one of the few studies that have been carried out to evaluate the activity of MBNPs loaded with an anti-inflammatory drug under in vivo conditions [68]. For this purpose, these authors prepared hollow mesoporous bioactive nanoparticles, which presented an ibuprofen loading efficiency between 29.5 and 55%, depending on the amount of surfactant added in the synthesis process. When these MBNPs were implanted in a tibia defect in rats, the group with the ibuprofen-loaded nanoparticles showed greater bone formation and less presence of inflammatory cells compared to the group with MBNPs without drug. This study was thus able to demonstrate that the incorporation of ibuprofen into this type of nanoparticle can promote osteogenesis and greatly alleviate inflammation. Table 2 summarizes some of the most significant drug delivery systems prepared with MBNPs so far.

## 5. Ion-Doped Mesoporous Bioactive Nanoparticles

In the last decade, some research groups have made important advances to provide MBNPs with new biological properties through the incorporation of different ions in the form of oxides within the SiO_2_-CaO-P_2_O_5_ system. Thus, MBNPs have been endowed with angiogenic, antitumor, anti-inflammatory, and antibacterial properties by incorporating different elements, most of which are metals from the major groups as well as from the d-block and, to a lesser extent, from the inner transition elements (rare earths) of the periodic table. Table 3 shows some of the most significant ions/oxides incorporated into MBNPs for therapeutic purposes.

The incorporation of boron into silica-based bioactive glasses leads to an increase in solubility, as well as to a faster transformation of borosilicates into hydroxyapatite. This is explained by the fact that [BO_3_] units increase the solubility of borosilicates. Zheng et al. [78] have studied the incorporation of B_2_O_3_ in amounts of less than 7% (% mol) in mesoporous nanoparticles of the binary system SiO_2_-CaO. These authors were able to verify that the incorporation of boron in these MBNPs did not influence the morphology of the particles. Studies with macrophages showed that the presence of boron led to a down-regulation of the expression of proinflammatory genes, which could have a potential immunomodulatory effect on the inflammatory response, although it also down-regulated the expression of pro-osteogenic genes in SaOS2. On the other hand, Bai et al. [79] demonstrated that the presence of boron in MBNPs of the SiO_2_-P_2_O_5_-CaO ternary system increased the amount of hydroxyapatite deposited under in vitro conditions, as well as had a positive effect on the proliferation of human periodontal ligament cells. The ab initio molecular dynamics (AIMD) simulations performed by these authors made it possible to relate the structural changes introduced by B_2_O_3_, in terms of the coordination number of boron atoms, to the improvement of in vitro bioactivity.

Among the different ions with potential therapeutic properties, the Zn^2+^ ion has been one of the most studied for incorporation into bioactive ceramics [80,81]. Zn^2+^ ions stimulate the differentiation of osteoprogenitor cells towards a mature osteoblast phenotype and have shown antibacterial properties under in vitro conditions. However, the presence of Zn^2+^ ions produces a decrease and even inhibits the crystallization of hydroxyapatite on the surface of MBNPs [82]. Thus, Zn^2+^ ions could favor osteogenic activity of MBNPs by stimulating osteoprogenitor differentiation [83], but, at the same time, inhibit this process by not allowing HA formation on the MBNPs’ surfaces. Zn-doped MBNPs have also been proposed to be part of organic–inorganic composites, included in PCL fibers for bone tissue regeneration [84] as well as as an additive in dental adhesives. In this context, Choi et al. [85] reported on the effects that the incorporation of Zn-doped MBNPs in dental adhesives had on the microtensile bond strength (MTBS) of this type of adhesives. These authors were able to demonstrate that the incorporation of 1.0% nanoparticles produced a significant increase in MTBS, although no significant differences were observed between MBNPs with and without Zn. On the other hand, dental adhesives containing Zn-doped MBNPs showed an increase in alkaline phosphatase as well as Alizarin Red upon contact with human dental pulp stem cells, indicating a substantial improvement in the remineralization capacity of this type of composite.

Vascularization of bone tissue is one of the most important aspects to consider for the development of osteogenic materials. In this regard, the Co^2+^ ion has shown angiogenic activity due to its property of mimicking hypoxia conditions in the organism. El-Fiqi and Kim reported the synthesis of MBNPs containing cobalt ions [86]. In their article, they stated that MBNPs could act as Co^2+^ ion release systems, so that at low concentrations they could have angiogenic activity, while for high Co^2+^ concentrations they could be potentially useful in the treatment of cancer by ferroptosis killing of cancer cells.

The incorporation of ions with antibacterial activity is one of the research lines that has aroused most interest in the field of MBNPs. The prophylaxis and treatment of bone infections can be addressed with the intraosseous administration of MBNPs doped with certain ions, offering an alternative to the administration of antibiotics. In this context, different researchers have proposed the incorporation of copper ions into MBNPs as active agents against Gram+ and Gram– bacteria, developing mesoporous bioactive nanoparticles in the systems SiO_2_-CaO-CuO [87] and SiO_2_-CaO-P_2_O_5_-CuO [88] while keeping their bioactive behavior under in vitro conditions. Among the challenges in the design of these systems are the aggregation of particles, the formation of copper nanoparticles, and the limited amount of copper ions that can be incorporated. Prof. Boccaccini’s group proposed the preparation of Cu-MBNPs using a Cu/ascorbic acid complex as precursor, as opposed to inorganic salts such as copper chloride or copper nitrate, frequently used in the synthesis of these nanosystems [89]. By means of this strategy, Cu-MBNPs with chemical homogeneity, highly dispersed, and with CuO amounts up to 6% mol were obtained, avoiding in turn the formation of Cu nanoparticles.

Silver and silver ions have been other species widely considered for their broad-spectrum antibacterial activity [90]. Zheng et al. [91] used the post-modification method to incorporate Ag^+^ ions into MBNPs of the SiO_2_-CaO system. This method consists of initially preparing SiO_2_-CaO MBNPs by the sol–gel method and then impregnating them with a silver nitrate solution, followed by heat treatment to decompose the nitrates. This avoids the occurrence of agglomerates that usually occur in one-step methods. These Ag-MBNPs showed antibacterial activity against *S. aureus* and *P. aeruginosa* in the planktonic state. However, when these authors studied their efficacy with an infected skin model, they observed that Ag-MBNPs were only effective against *S. aureus*, inhibiting activity against *P. aeruginosa*. This fact is explained by the more invasive nature of *P. aeruginosa* in the skin model, as this pathogen can penetrate deeper into the skin layers, avoiding contact with Ag-MBNPs. This study highlights the importance of designing models that mimic in some way the interactions that take place under in vivo conditions, as opposed to planktonic bacterial models in vitro.

One of the most interesting strategies consists of incorporating Ag^+^ cations together with other ions to enhance the antimicrobial effect or to provide MBNPs with multifunctionality. Leng et al. introduced Ag^+^ and Zn^2+^ into mesoporous SiO_2_-CaO-based nanoparticles showing that this system presents good activity against *E. faecalis* bacterial biofilm [92]. This effect is mainly due to the release of Ag^+^ ions, while the presence of zinc slightly stimulates cell proliferation, showing a potential application as a disinfectant for the root canal and dentinal tubules [93]. Similarly, the incorporation of Ag^+^ ions with Sr^2+^ in the chemical composition of MBNPs has been performed by Ur Rehman’s group [94], with the aim of combining the antimicrobial activity of Ag^+^ ions with the osteogenic activity of Sr^2+^. These nanoparticles showed activity against S. carnosus and E. coli, also being able to develop an apatite-like phase in contact with simulated body fluid. In a second work, the same researchers incorporated Ag-Sr-MBNPs into chitosan and gelatin coatings for orthopedic implants [95]. These coatings were deposited by electrophoretic deposition (EPD) on 316L stainless steel. By means of optimizing the EPD parameters, such as voltage, deposition time, and distance between electrodes, these authors obtained coatings with suitable adhesion to the substrate. In addition, the incorporation of Ag-Sr-MBNPs provided apatite-forming ability upon immersion in SBF as well as antibacterial activity against *E. coli*.

The incorporation of chalcogenide elements such as selenium and telluride into MBNPs has provided these compounds with antitumor activity. MBNPs of composition 60SiO_2_/36-CaO/4P_2_O_5/_xSeO_2_ have shown antitumor activity under in vitro conditions in cell cultures [96]. These nanospheres exhibited a specific inhibitory effect on MG63 osteosarcoma cells but caused minimal damage to normal bone cells (MC3T3-E1). On the other hand, the preparation of MBNPs containing telluride showed that incorporation of this element also shows activity against MG63 cells, due to its ability to promote apoptosis by an ROS-mediated mechanism [97]. Additionally, the presence of telluride as TeO_2_ did not affect the mineralization and degradation of the MBG nanoparticles.

Cations of rare earth elements, more specifically of the lanthanide series, have been of interest as additives to provide multifunctionality to MBNPs. Different research groups have studied the inclusion of cerium ions in the chemical composition of MBNPs [98,99,100,101]. The incorporation of these ions with oxidation states (III) and (IV) is justified by the anti-inflammatory activity of these species. Cerium readily exchanges its oxidation states between Ce^3+^ and Ce^4+^ under physiological conditions, which allows it to neutralize free radicals, including reactive oxygen species (ROS) [100], inducing anti-inflammatory effects. In this context, MBNPs have been prepared in the SiO_2_-CaO-Ce_2_O_3_ system, and their anti-inflammatory effect has been evaluated with RAW 264.7 macrophage cell cultures, demonstrating that the presence of cerium decreases the release of nitric oxide, indicative of the anti-inflammatory response against macrophages [98]. This potential anti-inflammatory effect of cerium-doped MBNPs has also been observed by reducing the expression of IL-1β and IL-6 as well as by decreasing the expression of oxidative-stress-related genes [99]. In parallel, the incorporation of cerium ions has shown an increase in the osteogenic potential of BMPNs, although all the studies performed so far have been in vitro conditions with different cell cultures [99,101].

The incorporation of Eu into nanoparticles of SiO_2_-CaO-P_2_O_5_-Eu_2_O_3_ composition used as doxorubicin carrier matrices showed that the presence of Eu improved drug loading capacity as well as drug release. In this regard, DOX-loaded Eu-loaded MBNPs maintain a long-term inhibitory effect on the viability of osteosarcoma MB63 cells [102], as well as on Hela cells [103]. Similarly, it has been possible to observe that incorporating appropriate Tb^3+^ ions into MBNPs significantly decreased doxorubicin release rate across the range of pH environments, indicating the role that these lanthanide elements may play in the control of antitumor drugs from MBNPs [104].

**Table 3:** Ion-doped mesoporous bioactive nanoparticles: compositions and therapeutic effects.

Ion/Oxide	Precursor	MBNP Composition	Biological Effect	Ref.
B^3+^/B_2_O_3_	H_3_BO_3_	50SiO_2_-(50−x)CaO-xB_2_O_3_ x = 10 and 15	Anti-inflammatory	[78]
		(60−x)SiO_2_-xB_2_O_3_-30.2CaO-9.8P_2_O_5_ (x = 0, 5, 10, 20)	Periodontal ligament cells proliferation	[79]
Zn^2+^/ZnO	Zn(NO_3_)_2_·6H_2_O	70SiO_2_-25CaO-5ZnO	Anti-inflammatory Pro-osteogenic	[82,83,105]
		60SiO_2_-31CaO-5P_2_O_5_-5ZnO	Remineralization of dental adhesives	[85]
		84SiO_2_-8CaO-8ZnO	Nanocomposite component	[84]
Co^2+^/CoO	Co(NO_3_)_2_·6H_2_O	85SiO_2_-(15−x)CaO-xCoO x = 0, 1 and 4	Angiogenesis Antitumoral	[86]
Cu^2+^/CuO	Cu/ascorbic acid complex	85.4SiO_2_-10.2CaO-4.4CuO	Improvement of mechanical properties of dental composites	[89,106]
	CuCl_2_	85SiO_2_-(15−x)CaO-xCuO x = 2 and 5	Antibacterial	[87]
	Cu(NO_3_)_2_	79.5SiO_2_-(18−x)CaO-2.5P_2_O_5_-xCuO x = 0, 2.5 or 5	Antibacterial	[88]
		70SiO_2_-25CaO-5CuO	ALP increase	[105]
Sr^2+^/SrO	SrCl_2_	85SiO_2_-11CaO-4SrO	Non-tested	[107]
	Sr(NO_3_)_2_	80SiO_2_-10CaO-5SrO-5P_2_O_5_ 80SiO_2_-10CaO-10SrO	Proliferation of ADSCs and WJSCs	[108]
	SrCl_2_	51SiO_2_-18CaO-20Na_2_O-4P_2_O_5_-7SrO	Osteogenic	[109]
Sr^2+^/SrO and Cu^2+^/CuO	SrCl_2_ CuCl_2_	85SiO_2_-13CaO-1SrO-1CuO	Non-tested	[110]
Mg^2+^/MgO	MgCl_2_·6H_2_O	51SiO_2_-18CaO-20Na_2_O-4P_2_O_5_-7MgO	Non-tested	[111]
	Mg(NO_3_)_2_·6H_2_O	85SiO_2_-(15−x)CaO-xMgO	Non-tested	[112]
Mn^2+^/MnO	MnCl_2_·4H_2_O	50SiO_2_-(40−x)CaO-xMnO-10P_2_O_5_	Antibacterial Osteogenic	[113,114,115]
		70SiO_2_-25CaO-5MnO	Pro-osteogenic	[105]
Ag^+^/Ag_2_O	AgNO_3_	87SiO_2_-10.4CaO-2.6Ag_2_O	Antibacterial	[91]
Ag^+^/Ag_2_O and Zn^2+^/ZnO	AgNO_3_ and Zn(NO_3_)_2_·6H_2_O	(26–27.4)Si-(2.4–5.9)Ca-(0–5.2)Ag-(0–12.4)Zn (elemental wt%)	Antibacterial	[92,93]
Ag^+^/Ag_2_O and Sr^2+^/SrO	AgNO_3_ and Sr(NO_3_)_2_	70SiO_2_-24CaO-5SrO-1Ag_2_O	Antibacterial Improved bioactivity	[94]
		50SiO_2_-10P_2_O_5_-34CaO-5SrO-1Ag_2_O	Antibacterial	[95]
Se^4+^/SeO_2_	Na_2_SeO_3_	60SiO_2_-36-CaO-4P_2_O_5_-xSeO_2_	Antitumoral	[96]
Te^4+^/TeO_2_	Na_2_TeO_3_	60SiO_2_-36-CaO-4P_2_O_5_-xTeO_2_	Antibacterial Antitumoral	[97]
Ce^3+^/Ce_2_O_3_ and/or Ce^4+^/CeO_2_	Ce(NO_3_)_3_·6H_2_O	70SiO_2_-29CaO-1Ce_2_O_3_	Anti-inflammatory Antibacterial	[98]
		86SiO_2_-12CaO-2CeO_2_	Anti-inflammatory Pro-osteogenic	[99]
		85SiO_2_-(15−x)CaO-xCe_2_O_3_ x = 0, 5 and 10	ROS scavenger	[100]
		86.6SiO_2_-12.1CaO-1.3CeO_2_ 86.0SiO_2_-11.8CaO-2.2CeO_2_	BMSC differentiation Development of ECM	[101]
Eu^3+^/Eu_2_O_3_	Eu_2_O_3_	60SiO_2_-(36−x)-CaO-xEu_2_O_3_-P_2_O_5_ (x = 0, 0.5, 1, 2 mol%)	Controlled drug release	[102]
	Eu(NO_3_)_3_·6H_2_O	SiO_2_-CaO-P_2_O_5_-Eu_2_O_3_	Cancer diagnosis and treatment	[103]
Tb^3+^/Tb_2_O_3_	Tb(NO_3_)_3_·5H_2_O	79.5SiO_2_-15CaO-5P_2_O_5_-0.5Tb_2_O_3_	Controlled drug release	[104]

## 6. Specific Cell Response to Mesoporous Bioactive Nanoparticles

The ability of MBNPs to internalize in bone cells and generate specific responses can be used as a strategy for the treatment of bone pathologies. In this sense, MBNPs have high potential for treatment of osteoporotic bone due to their specific characteristics and the possibility of loading anti-osteoporotic drugs into their pores to be released into bone cells after intracellular uptake [116].

Different drugs can be used to treat osteoporotic bone, either by stimulating osteogenesis or by inhibiting the osteoclast resorptive activity, or both [117]. These anti-osteoporotic drugs include ipriflavone (IP), a synthetic isoflavone that inhibits bone resorption, maintains bone density, and prevents osteoporosis [118]. The administration of IP loaded into nanoparticles (MBNP/IPs) for intracellular release is a very interesting strategy, since the amount of IP required to improve the function of bone cells would be much lower. Prior to in vivo implantation, the impact of MBNP/IPs on specialized cell types involved in bone remodeling, osteogenesis, angiogenesis, and immune response has recently been evaluated by our group with satisfactory results.

**Figure 4:**
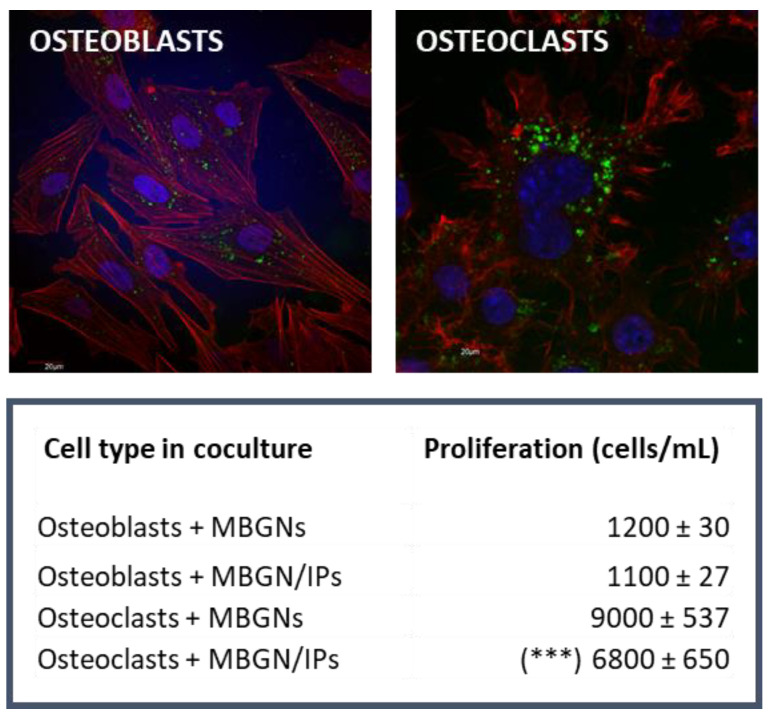
Incorporation of MBGNs labelled with FITC by osteoblasts/osteoclasts after 7 days in coculture and effects of MBGNs and MBGN/IPs on the proliferation of each cell type. Confocal images show the intracellular FITC-MBGNs in green, the actin cytoskeleton in red, and the nuclei in blue. Statistical significance: *** *p* < 0.005, comparison between osteoclasts treated with MBGNs and MBGN/IPs.

**Figure 5:**
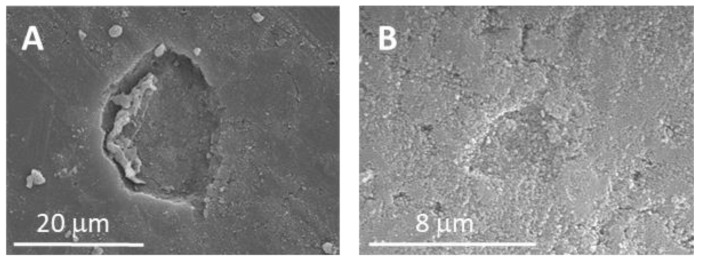
Resorption cavities left by osteoclasts on hydroxyapatite disks after 7 days in coculture with osteoblasts in the presence of 50 μg/mL of either MBGNs (**A**) or MBGN/IPs (**B**) observed by scanning electron microscopy.

Bone remodeling requires the concerted action of bone-forming osteoblasts and bone-resorbing osteoclasts [119], which communicate with each other through mechanisms that involve the release of different cytokines, extracellular matrix interactions, and cell–cell contact [120]. The use of cocultures of osteoblasts and osteoclasts as an experimental model allowed us to investigate in vitro the specific response of each of these two cell types after nanoparticle incorporation in conditions closer to the physiological situation. Thus, we were able to observe that the intracellular incorporation of MBNP/IPs induced a significant decrease in osteoclast proliferation and resorption activity after 7 days in coculture with osteoblasts, without affecting osteoblast proliferation and viability (Figure 4). Drug release tests demonstrated that only a fraction of the payload is delivered by diffusion, whereas the rest of the drug remains within the hollow core after 7 days, thus ensuring local long-term pharmacological treatment beyond the initial fast IP release [23]. By observing the resorption cavities left by osteoclasts on hydroxyapatite, we could demonstrate that, in the presence of IP-loaded MBGNs, osteoclasts in coculture exhibited a lower resorptive activity (Figure 5).

In addition, our studies with osteoprogenitor cells have shown that the active incorporation of MBGN/IPs by MC3T3-E1 pre-osteoblasts stimulates their differentiation into mature osteoblast phenotypes with increased alkaline phosphatase activity [121]. This cell line represents the most relevant model of in vitro osteogenesis [122]. The results have also indicated that these nanoparticles enter the pre-osteoblasts mainly through clathrin-dependent mechanisms and in a lower proportion by macropinocytosis.

The evaluation of the specific responses of osteoclasts, osteoblasts, and pre-osteoblasts to mesoporous bioactive glass nanoparticles loaded with IP demonstrates the great potential of this nanomaterial to stimulate osteogenesis and to inhibit bone resorption.

Taking into account the essential role of blood vessels in supplying oxygen and nutrients to ensure the development and repair of all tissues [123], the effects of MBGNs and MBGN/IPs on the cells involved in the angiogenesis process is another important aspect to assess. The use of endothelial progenitor cells (EPCs), directly involved in the formation of blood vessels, offers great advantages as an experimental model [124]. The specific response of EPCs to MBGN/IPs has recently been studied by our group, analyzing the expression of vascular endothelial growth factor receptor 2 (VEGFR2) [125]. This receptor is the major signal transducer for angiogenesis through the PLCγ –PKC–MAPK pathway [126]. Our studies evidenced that, after their incorporation by EPCs, MBGNs and MBGN/IPs promote the expression of VEGFR2, directly involved in angiogenesis, even in the presence of macrophages. These nanoparticles enter the EPCs through clathrin-dependent endocytosis, phagocytosis, and caveolae-mediated mechanisms.

The impact of MBGNs on the immune system has recently been addressed by evaluating the specific responses of different mouse immune cells, including spleen cell subsets, bone-marrow-derived dendritic cells (BMDCs), and cell lines like RAW 264.7 macrophages, SR.D10 Th2 CD4^+^ lymphocytes, and DC2.4 dendritic cells. After their rapid incorporation into T and B lymphocytes or specialized antigen-presenting cells (APCs) like dendritic cells (DCs) cells, MBGNs did not trigger cytotoxic responses, pro- and anti-inflammatory cytokines production, or T cell activation [127]. This study also demonstrated that these nanoparticles did not alter the maturation of BMDCs or DC2.4 cells, as shown by expression of major histocompatibility complex (MHC) and costimulatory molecules (CD40, CD80, CD86), nor IL-6 secretion.

In this context, it is important to highlight that macrophages are professional phagocytic cells which play a central role in host defense and are responsible for in vivo nanoparticle trafficking [128]. Macrophages have functional plasticity that allows them to switch between M1 pro-inflammatory and M2 anti-inflammatory phenotypes, characterized by the secretion of different cytokines and the expression of specific cell surface markers [129]. These phenotypes are crucial in inflammation/healing processes, and, for this reason, the evaluation of the in vitro polarization of macrophages after exposure to nanomaterials is important to know the potential in vivo host response [130]. Our studies with macrophages have evidenced that MBGNs did not induce macrophage polarization towards the M1 pro-inflammatory phenotype, favoring the M2 reparative phenotype [131].

On the other hand, phagocytosis by macrophages is necessary for the degradation of infectious agents and senescent cells, and it is involved in inflammation and tissue remodeling mechanisms [132]. Alteration of macrophage phagocytic ability is critical in the progression of different diseases [133] because this produces a suppressive adaptive immune response [134]. Thus, evaluation of macrophage defense capability against pathogens after nanoparticle uptake is necessary to know the nanomaterial’s effects on this specific macrophage function. In this context, we have recently evidenced that the intracellular uptake of MBGNs did not alter macrophage immunocompetence against Candida albicans and induced a transitory increase in fungal phagocytosis by macrophages after a short time of interaction [135]. The results also confirmed that macrophages clearly distinguish between inert material and the fungus in a dynamic intracellular incorporation.

In conclusion, studies of specific cell responses to MBGNs and MBGN/IPs carried out with cell types involved in bone remodeling, osteogenesis, angiogenesis, and immune response evidence the high potential of these nanoparticles for use as drug delivery systems in bone repair and osteoporosis treatments.

## 7. In Vivo Studies

The high potential of the mesoporous bioactive glass nanoparticles (MBGNs) as drug carriers and repair biomaterials for bone tissue regeneration is very evident, and this is due to their unique structure, properties, and biocompatibility demonstrated in vitro. However, it is important to consider that the application of any type of nanoparticle in vivo requires knowledge of its biodistribution, half-life in blood circulation, clearance, and possible toxicity in tissues and organs [136,137].

Regarding MBGN systemic metabolism and biosafety, B. Sui et al. used isotope quantitative tracing combined with biochemical parameters and histopathological changes to analyze the biodistribution, excretion, and effects on metabolism and major organs of these nanoparticles labeled with ^45^Ca. In their study, a suspension with 1 mg/mL of ^45^Ca-MBGNs was prepared in physiological saline and injected intravenously in mice via tail vein in a dose of 10 mg/kg body weight. The results indicated that, after cleared from circulation, these nanoparticles were mainly in the liver and localized in the cytoplasm of hepatocytes. Although some nanoparticles were in the mitochondria, this did not increase the intracellular content of reactive oxygen species (ROS). Furthermore, no abnormal metabolism and histopathological changes were observed. The accumulations of MBGNs in various organs were excreted mainly through feces. This study revealed that these nanospheres have no obvious biological risk and supported their use as drug delivery systems in clinical applications [138].

Mao et al. examined the acute toxicity and biodistribution of intravenously administrated monodispersed sub-micrometer mesoporous bioactive glass spheres in ICR mice. The lethal dose 50 (LD50) of these particles was higher than 250 mg/kg. The acute toxicity was evaluated at 14 days after intravenous injection of 20, 100, and 180 mg/kg in ICR mice through the tail vein, which is an extreme way of assessing the toxicity of particles that would normally be placed directly in the defect. The results revealed the low in vivo toxicity of these spheres at all doses. However, histological examination showed lymphocytic infiltration and granuloma formation in the liver and megakaryocyte hyperplasia in the spleen at high doses. Transmission electron microscopy studies indicated that these particles were mainly distributed in the resident macrophages of the liver and spleen and could be cleared from the body after more than 2 weeks [139].

Several studies have already been carried out with different experimental animal models to analyze the effects of the local application of mesoporous bioactive glass nanoparticles in vivo for different purposes. These in vivo studies include the implantation of MBGNs in different forms, such as free nanoparticles in suspension or as part of cements and scaffolds. In this context, hollow mesoporous bioglass nanoparticles loaded with or without ibuprofen (IBU) and mixed with phosphate buffer were used to fill bone defects 3 mm diameter × 3 mm deep created on the tibias of ten-week-old Wistar rats in order to stimulate bone regeneration. After four weeks’ implantation, osteogenesis ability was evaluated by the method of hematoxylin and eosin staining and Masson’s trichrome staining. The results evidenced that MBGNs promoted osteogenesis while IBU-MBGNs facilitated bone regeneration and alleviated local inflammation at the same time [75].

**Figure 6:**
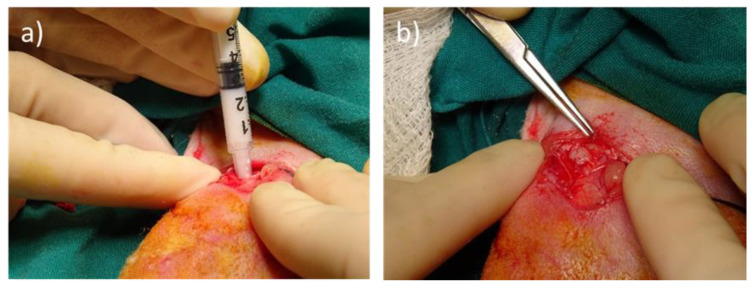
MBNP–hyaluronic acid implantation as an injectable material (**a**); Image of the defect filled with the gelled material (**b**).

Pontremoli et al. reported the use of MBGNs embedded into hydrogels as injectable platforms for the in situ delivery of therapeutic ions [140]. This strategy has very recently allowed us to deliver MBGNs as intraosseous biomaterials via minimally invasive surgery in an osteoporotic animal model to evaluate the bone regenerative capability of both MBGNs and MBGNs loaded with ipriflavone as an anti-osteoporotic drug (MBGN/IPs) [141]. Previously, our research group had widely studied the in vitro specific cell responses to MBGNs and MBGN/IPs with cell types involved in bone remodeling, osteogenesis, angiogenesis, and immune response, evidencing the high potential of these nanoparticles for use as drug delivery systems in bone repair and osteoporosis treatments [23,121,125]. In our very recent in vivo studies, MBNPs and MBGN/IPs, suspended in a hyaluronic-acid-based hydrogel, were injected intraosseously into cavitary bone defects in osteoporotic rabbits obtaining successful results (Figure 6). Thus, MBNPs promoted bone regeneration with a moderate inflammatory response, as histological analyses have evidenced. On the other hand, MBGN/IPs produced a higher presence of osteoblasts and enhanced angiogenesis at the defect site, but without showing significant differences in terms of new bone formation [141].

Another interesting objective of MBGN design is the local treatment of tumors with anti-cancer-drug-loaded MBGNs. In this context, dendritic MBGNs with antitumor activity and controlled drug release have been successfully achieved and evaluated against an in vivo mouse tumor xenograft model. These nanoparticles were loaded with doxorubicin (DOX) and did not induce pathological changes in major organs during treatment, but produced cell lysis, fragmentation, and reduction in tumors. However, animals treated with free DOX exhibited serious side effects, including interstitial lymphocytes infiltrated into the heart, which may be related to drug-induced myocarditis. Thus, dendritic MBGNs could be used to synergize with anti-cancer drugs, improving antitumor efficacy and reducing systemic toxicity [59].

On the other hand, many strategies have also been carried out to prepare nanocements from mesoporous bioactive glass nanoparticles, improving their properties as promising injectable materials for bone repair and regeneration. In this sense, M.S. Kang et al. [142] used MBGNs with different compositions (Ca content varied widely from 0 to 25%) and mesopore sizes (small and large pores) to obtain nanocements, analyzing the apatite-forming ability, the protein adsorption capacity, the ionic releases, and the ability of early bone induction in a rat calvarium defect. In this study, different techniques (TEM, XRD, FT-IR, XPS, and NMR) were used and demonstrated that the cementation mechanism was ionic (Si and Ca) dissolution and then reprecipitation to form Si-Ca-(P)-based amorphous nano-islands that could network the particles. The release of ions significantly stimulated the responses of the cells studied (rMSCs and HUVECs), enhancing rMSC viability and osteogenesis as well as HUVEC tubular networking. The nanocement also increased in vivo neo-blood-vessel formation in a chorioallantoic membrane (CAM) model, evidencing that Si ion release might play a significant role in pro-angiogenesis. Furthermore, nanocement implantation in a rat calvarial bone defect produced signs of osteoinductivity along with excellent osteocondution and bone matrix formation.

In this context, MBGNs are considered excellent nano-additives to calcium phosphate cements (CPCs) due to their nanoscale size (with high surface-area-to-volume ratio), spherical morphology (which allows homogenous mixing, good followability and better injectability), high specific surface area, and high mesoporosity (which increased surface bioreactivity and degradability) [143]. Thus, highly bioactive bone cement microspheres, based on α-tricalcium phosphate (α-TCP) microparticles and MBGN nanoparticles, have very recently been designed, characterized, and implanted in vivo for bone regeneration. The results obtained by histological analyses, X-ray radiography, and micro-computed tomography evaluations evidenced that the in vivo implantation of α-TCP/MBGN cement microspheres in rat calvarial critical-size bone defects induced high bioactivity and new bone ingrowth and formation after 6 weeks [144].

MBGNs combined with polymers have also been used for the preparation of scaffolds designed for bone repair. Thus, C. Covarrubias et al. [145] produced bionanocomposite scaffolds by incorporating dense bioactive glass nanoparticles or mesoporous bioactive glass nanospheres into a chitosan–gelatin polymer blend. These scaffold nanocomposites were evaluated in vivo using a critical-sized femoral defect model in Sprague-Dawley adult rats, demonstrating that, after eight weeks of implantation, bioactive glass nanoparticles of (5%)/chitosan–gelatin bionanocomposite significantly enhanced the amount of new bone (80%) in the defect area. This high bone regeneration capacity observed with the scaffolds prepared with MBGNs makes them very attractive for bone reconstruction applications.

## 8. Conclusions

MBNPs offer the possibility of obtaining drug and therapeutic ion delivery nanosystems with intrinsic osteogenic capacity. This aim has attracted the attention of different research groups to preparing implantable nanocomposites in bone tissue, additives in dental materials, and intra-osseous injectable systems for the treatment of pathologies such as osteoporosis, prosthetic infection, and bone tumors. The small size of MBNPs, which are mostly spherical in morphology with diameters between 50 and 200 nm, allows them to pass through biological barriers and be internalized by the different cell types found in bone tissue. This allows MBNPs to elicit specific cellular responses and can effectively modify essential processes such as bone remodelling and both specific and non-specific inflammatory responses. However, the small size is also responsible for the occurrence of toxic effects, since MBNPs can invade cells surrounding the implantation site and migrate through the bloodstream, causing further damage to distant tissues and organs. This is why the effort to develop different synthesis methodologies that maximize therapeutic effects while decreasing toxicity is one of the most important challenges facing this technology.

There is a broad consensus that the structural characteristics of MBNPs, such as large surface area, high pore volume, and easy chemical functionalization, are unique for their use in therapeutic, diagnostic, and theragnostic purposes. There are numerous studies and reviews that address these aspects of materials with certain similarities to MBNPs, such as mesoporous silica nanoparticles (MSNs), bioactive mesoporous glasses (MBGs), and nanoparticles obtained by the sol–gel method without including structure-directing agents. In this sense, it has been possible to demonstrate that the in vivo behavior of MSNs is closely related to the preparation method used, particle size, particle geometry, chemical properties, doses used, and routes of administration [146]. Additionally, different parameters, such as the morphology and size of the mesostructures, electrostatic interactions between MSNs, and cell membrane and surface functionalization, can affect the endocytic mechanisms of MSN uptake [147,148]. Another important aspect is that Si ions can be released in a gradual and controllable manner from MSNs by adjusting the specific surface area [149]. In the field of MBGs, the influence of the mesoporous structure on the proliferation and differentiation of osteoprogenitor cells and apatite-forming ability has been also demonstrated [150].

However, to the best of our knowledge, there are no studies of this nature in the specific field of MBNPs. Simplistically, MBNPs can be considered MSNs containing CaO and P_2_O_5_, or as nano-sized MBGs, but specific studies focused on the importance of the physicochemical properties of MBNPs for bone regeneration applications are needed to facilitate the translation of MBNPs to clinical practice.

On the other hand, despite the interest that MBNPs have aroused in the last decade, the number of in vivo studies demonstrating their therapeutic potential is very scarce. Similarly, very few experiments have dealt in depth with the potential toxicity of these nanomaterials in terms of their biodistribution depending on the route of administration. This is undoubtedly an essential aspect that should be investigated in depth, since the ability to release Ca^2+^ ions into the surrounding medium can greatly limit (or even prevent) the intravenous administration of MBNPs.

## Figures and Tables

**Table 2:** Drug delivery systems based on mesoporous bioactive nanoparticles.

Therapeutic Effect	Drug	MBNP System *	Ref.
Osteogenesis	Icariin	Silk fibroin/MBNPs scaffolds	[44]
	Icariin	Alginate dialdehyde–gelatin hydrogel/MBNPs scaffolds	[46]
	Dexamethasone	Polycaprolactone–gelatine/MBNPs scaffolds	[50]
	Phenamil	Sr doped MBNPs	[53]
	FGF2 + FGF8	Polyethylene oxide/polycaprolactone electrospun scaffolds containing MBNPs	[52]
Antitumoral	Doxorubicin	SiO_2_-CaO-P_2_O_5_ MBNPs	[22]
	Doxorubicin	SiO_2_-CaO hollow MBNPs	[24]
	Doxorubicin	SiO_2_-CaO-P_2_O_5_ MBNPs	[58]
	Doxorubicin	SiO_2_-CaO-P_2_O_5_ MBNPs	[59]
	Doxorubicin	SiO_2_-CaO-Na_2_O-P_2_O_5_-Ag_2_O MBNPs	[74]
	Imatinib	SiO_2_-CaO-Na_2_O-K_2_O-P_2_O_5_ MBNPs	[62]
	Daunomycin	Poly-L-glutamic acid embedded MBNPs	[61]
	Silibinin	SiO_2_-CaO MBNPs	[60]
	Fluorouracil	SiO_2_-CaO-P_2_O_5_ MBNPs	[71]
Antimicrobial	Boswellia sacra extract	APTES modified SiO_2_-CaO-P_2_O_5_ MBNPs	[69]
	Cinnamaldehide	MBNP/PHBV-based microspheres	[68]
	Vancomicin	Hollow MBNPs	[14]
	Triclosan	Ordered mesopores microspheres	[11]
	Ampicilin	APTES modified SiO_2_-CaO MBNPs	[66]
		SiO_2_-CaO MBNPs	[67]
Anti-inflammatory	Ibuprofen	SiO_2_-CaO MBNPs	[72]
		SiO_2_-CaO-P_2_O_5_ hollow MBNPs	[75]
	Curcumin	MBNP/PHBV-based microspheres	[73]
	Diclofenac	SiO_2_-CaO-P_2_O_5_ MBNPs	[71]
Analgesia	Ropivacaine	PLA-PEG coated MBNPs	[76]
Gene transfection	miRNA	SiO_2_-CaO-P_2_O_5_ MBNPs	[71]
	siRNA	APTES modified SiO_2_-CaO MBNPs	[66]
Drug model	BSA	SiO_2_-CaO-P_2_O_5_ MBNPs	[77]

* APTES: 3-amminopropyltriethoxysilane; PHBV: Polyhydroxybutyrate-co-hydroxyvalerate; PLA-PEG: polylactic acid-b-polyethylene glycol; BSA: bovine serum albumina.
